# Suizid und Suizidbeihilfe aus rechtlicher und ethischer Perspektive

**DOI:** 10.1007/s00103-021-03469-9

**Published:** 2021-12-17

**Authors:** Tanja Henking

**Affiliations:** Hochschule für angewandte Wissenschaften Würzburg-Schweinfurt, Münzstr. 12, 97070 Würzburg, Deutschland

**Keywords:** Beihilfe zum Suizid, Freiverantwortlichkeit, Autonomie, Lebensschutz, Recht auf selbstbestimmtes Sterben, Assisted suicide, Free responsibility, Autonomy, Life protection, Right to self-determined death

## Abstract

Das Bundesverfassungsgericht hat im Jahr 2020 das Verbot der geschäftsmäßigen Beihilfe zur Selbsttötung für verfassungswidrig und nichtig erklärt. Das Gericht leitet aus dem allgemeinen Persönlichkeitsrecht ein Recht auf selbstbestimmtes Sterben ab. Dieses Recht schließe auch die Freiheit ein, sich das Leben zu nehmen sowie hierfür bei Dritten Hilfe zu suchen und Hilfe, soweit sie angeboten wird, auch in Anspruch zu nehmen. Inzwischen liegen einige Regelungsvorschläge und Gesetzesentwürfe vor, die unterschiedliche Konzeptionen einer möglichen zukünftigen Regelung der Suizidbeihilfe verfolgen. Der Suche nach einer neuen Regelung sollte aber aus strafrechtlicher Perspektive stets die Frage nach der Erforderlichkeit einer neuen Regelung vorausgehen. Eine Neuregelung darf sich nicht auf bestimmte Personengruppen, wie beispielsweise Personen mit unheilbarer, tödlicher Erkrankung, beschränken, weil andernfalls das Suizidmotiv bewertet würde. Dieses bringt die besondere Herausforderung mit sich, eine Regelung zu finden, die ohne eine Bewertung des Suizidmotivs die unterschiedlichen Problem- und Bedürfnislagen erfasst und dabei auch in den Blick nimmt, dass die Autonomie des Einzelnen in unterschiedlicher Weise gefährdet sein kann.

Der Beitrag nimmt seinen Ausgangspunkt in dem Recht auf Selbsttötung, beleuchtet unterschiedliche Konzepte und diskutiert ihre Vor- und Nachteile, ohne einzelne Gesetzesvorschläge explizit herauszustellen. Hierdurch soll die weitere Debatte um einzelne Aspekte bereichert werden. Zugleich wird dafür geworben, gesetzgeberische Zurückhaltung zu üben.

## Abstract (en)

In 2020, the Federal Constitutional Court declared the ban on assisted suicide unconstitutional and invalid. The court derived a right to self-determined dying from the general right of personality. This right also includes the freedom to take one’s own life and to seek help from third parties for this purpose and to make use of help if it is offered. In the meantime, there are several proposals for regulations and draft laws that pursue different concepts of a possible future regulation of assisted suicide. However, from the perspective of criminal law, the search for a new regulation should always be preceded by the question of the necessity of a new regulation. A new regulation must not be limited to certain groups of persons, such as persons with incurable, terminal illnesses, because otherwise the suicide motive would be assessed. This brings with it the particular challenge of finding a regulation that covers the different problem and need situations without assessing the suicide motive and also takes into account that the autonomy of the individual can be endangered in different ways.

The article takes its starting point in the right to suicide, sheds light on different concepts, and discusses their advantages and disadvantages without explicitly highlighting individual legislative proposals. This is intended to enrich the further debate with individual aspects. At the same time, it advocates legislative restraint.

## Einleitung

Entscheidend für die strafrechtliche Beurteilung, ob eine straflose „Beihilfe zur Selbsttötung“ vorliegt oder eine strafbare Handlung („Totschlag in mittelbarer Täterschaft“), ist die Frage, ob der Suizid auf einer freiverantwortlichen Entscheidung des Suizidenten beruht.^1^ Aus der Perspektive der Suizidprävention folgt hieraus allerdings nicht, dass Suizidprävention nicht auch bei dem freiverantwortlichen Suizidwunsch anzusetzen hat und auch diesen verhindern will. Hier verläuft eine für die Debatte wichtige Linie: Es ist moralisch keineswegs verwerflich, eine Person von einem Suizid abzuhalten, sie zum Leben überreden zu wollen, gleichwohl sie ein Recht auf selbstbestimmtes Sterben einschließlich der Selbsttötung hat. Im Gegenteil: Intuitiv werden die meisten Menschen dieses als moralisch richtiges Handeln bezeichnen. Doch: Bleibt es beim freiverantwortlichen Entschluss, dann ist diese Entscheidung zu akzeptieren.

Ob man selbst zur Hilfe bei der Umsetzung des Entschlusses bereit wäre, wird vom eigenen moralischen Wertesystem sowie von weiteren Umständen abhängen. Einfluss hierauf nehmen könnte die Nachvollziehbarkeit des Motivs für den Suizidwunsch. So mag für den einen der Wunsch bei unheilbarer, tödlicher Erkrankung nachvollziehbar sein, für den anderen bei psychischen Erkrankungen und ein Dritter trifft womöglich keine Differenzierung. Dies mögen aus der Perspektive des Einzelnen nachvollziehbare Überlegungen sein, doch steht es uns eigentlich zu, den Suizidwunsch einer Person und deren Motiv moralisch zu bewerten? Die Antwort des Bundesverfassungsgerichts (BVerfG) könnte deutlicher nicht ausfallen: Dem Staat und der Gesellschaft steht dies nicht zu. Die Entscheidung des BVerfG (153, 182 ff.) aus dem Jahr 2020 führt eine jahrelange Debatte fort, hat Eckpfeiler gesetzt und gleichzeitig weitere Fragen aufgeworfen:

- Soll die Beihilfe zur Selbsttötung rechtlich geregelt werden? Wenn Ja, in welcher Form und zu welchem Zweck? Als Schlagworte können aufgerufen werden: Schutz vor Missbrauch, Sicherung der Freiverantwortlichkeit, Zugang zur Suizidbeihilfe, Rechtssicherheit für die Beteiligten.
- Wer soll Beihilfe zum Suizid leisten (dürfen)? Wie geht man damit um, dass Suizidmotive unterschiedlich ausfallen, unterschiedliche Personengruppen eine Hilfe zur Selbsttötung begehren können, der Staat diese Motive aber nicht bewerten und damit einzelne Gruppen aus der Suizidbeihilfe nicht exkludieren darf?
- Wie kann gewährleistet werden, dass der Suizidentschluss freiverantwortlich erfolgt, die Person alle für sie wichtigen Informationen zur Entscheidung erhält, sie Alternativen kennt, ihr adäquate Angebote unterbreitet wurden? Wie verhalten sich Freiverantwortlichkeit und die Ambivalenz eines Sterbewunsches zueinander?
- Und letztlich die herausfordernde Frage: Wie setzen wir dieses alles um, ohne in eine Art „Regelungseifer“ zu verfallen und eine existenzielle, höchst persönliche Entscheidung zu stark zu formalisieren? Laufen wir hier Gefahr vulnerable Gruppen gegeneinander auszuspielen?

## Suizid: kein (strafbares) Unrecht

Während die Diskussion um den richtigen Umgang mit dem Sterbewunsch einer Person seit jeher kontroverse Debatten auslöst, besteht über eine rechtliche Frage weitgehend Konsens: Der Suizid ist nicht strafbar [1–6]. Darüber, dass der Suizid kein Unrecht darstellt oder, wie in der Rechtsprechung zuweilen behauptet wurde, eine selbstherrliche Verfügung über das eigene Leben ist, besteht ebenfalls ein großer Konsens in der rechtswissenschaftlichen Debatte. Wenn der Suizid kein Unrecht darstellt, dann leistet der Helfende auch keine Unrechtshilfe [2, 3, 5]. Will man die Suizidbeihilfe nun also (wieder) unter Strafe stellen, wie es aktuelle Gesetzesentwürfe bzw. -vorschläge teilweise vorsehen, bedarf es einer Begründung, warum die jeweilige Form der Beihilfe nun (ein selbstständiges) Unrecht sei.

Eine moralische Bewertung des Suizids ist in einer rechtlichen Debatte deplatziert [5, 7], weil der Staat kein Recht hat, die Handlung und die Motive des Einzelnen zu bewerten. Welchen Bewertungsmaßstab sollte er auch anlegen, ohne die Vielfalt von Wertvorstellungen einer pluralistischen Gesellschaft zu negieren? Jeder Versuch, erneut mit einer Strafnorm zu arbeiten, wird sich also der Frage stellen müssen, warum beispielsweise ein gewerbsmäßiges oder geschäftsmäßiges Handeln (bzgl. Suizidbeihilfe) strafbares Unrecht darstellt.

Durchaus uneinheitlich wurde die Frage diskutiert, ob der Einzelne auch ein Recht zur Selbsttötung habe. Diese Frage ist durch das BVerfG-Urteil geklärt: Danach umfasst das allgemeine Persönlichkeitsrecht (Art. 2 Abs. 1 i. V. m. Art. 1 Abs. 1 Grundgesetz) als Ausdruck persönlicher Autonomie ein Recht auf selbstbestimmtes Sterben. Das Recht auf selbstbestimmtes Sterben schließt die Freiheit ein, sich das Leben zu nehmen sowie hierfür bei Dritten Hilfe zu suchen und Hilfe, soweit sie angeboten wird, in Anspruch zu nehmen.

Bemerkenswert ist dabei die klare Absage an jeglichen Versuch, den Suizid – moralisch – zu bewerten. So sei die Entscheidung des Einzelnen, seinem Leben entsprechend seinem Verständnis von Lebensqualität und Sinnhaftigkeit der eigenen Existenz ein Ende zu setzen, als Akt autonomer Selbstbestimmung von Staat und Gesellschaft zu respektieren (BVerfGE 153, 182, 2. Leitsatz).

Die Frage, ob ein Suizid „falsch“ ist, sei es grundsätzlich oder manchmal, stellt sich aus rechtlicher Perspektive nicht. Die philosophische Debatte um die ethische Betrachtung des Suizids dürfte ganze Bücherregale füllen [8]. Im Kern geht es der rechtlichen Debatte um eines: Darf der Staat den Suizid bzw. die Beihilfe zum Suizid verbieten? Lässt sich ein strafbares Unrecht feststellen, das ein Verbot rechtfertigen könnte? Ausgehend von der Vorstellung, dass jeder Mensch sein Leben frei gestalten kann und sein Leben so lebt, wie es dem eigenen Wertesystem entspricht, führt dieses unweigerlich zu dem Schluss, dass der Mensch sein Leben auch frei beenden kann [9–11].

Der Staat darf nicht, zudem nicht mit seinem schärfsten Schwert dem Strafrecht, dem Einzelnen die Freiheit zur Selbsttötung durch die faktisch fehlende Möglichkeit der Inanspruchnahme Dritter nehmen. Das hier angesprochene Respektieren der Entscheidung des Einzelnen bedeutet zugleich die Zurückhaltung vor einer moralischen Bewertung der Selbsttötung. So finden sich in der Entscheidung mehrere Passagen, die darauf hinweisen, dass die Selbsttötung und die Inanspruchnahme Dritter nicht abgewertet werden dürfen. So verfolge ein Gesetz keinen legitimen Zweck, wenn es „die Entscheidung des mit autonomem Willen handelnden Grundrechtsträgers, sich mit der Unterstützung Dritter bewusst und gewollt selbst zu töten, als solche missbilligt, tabuisiert oder mit einem Makel belegt“ (BVerfGE 153, 182, Rn. 234).

Die Bundesärztekammer hat jüngst erneut einen anderen Weg eingeschlagen [12]. In der beschlossenen Änderung der Musterberufsordnung heißt es nun, dass die Beihilfe zur Selbsttötung „keine ärztliche Aufgabe“ sei. Belegt sie mit dieser Formulierung die Beihilfe zum Suizid nicht doch mit einem Makel? Der Beistand bis zuletzt ist eine ärztliche Aufgabe, der Beistand bis zum selbstbestimmten Zuletzt jedoch nicht?

## Notwendigkeit einer Regelung?

Das BVerfG hat bekanntlich die Regelung des § 217 StGB^2^ (geschäftsmäßige Förderung der Selbsttötung) für verfassungswidrig und nichtig erklärt. Dabei hat es den Weg für eine gesetzliche Neuregelung nicht versperrt, sondern vielmehr auf die Bandbreite denkbarer Regelungsansätze hingewiesen. Inzwischen sprechen sich sogar einige für eine Regelung aus, die zuvor teils vehement gegen eine Strafbarkeit der Suizidbeihilfe argumentiert haben [10, 13]. Es überrascht, wenn nun von Gefahren der Manipulation oder des Missbrauchs gesprochen wird. Sind die Gefahren größer geworden als sie es noch 2015 waren? Handelt es sich um eine (neue) Gefahr, wenn z. B. auch Sterbehilfevereine nun wissen, dass ihr Handeln nicht (mehr) verboten ist und sie nun uneingeschränkt tätig werden können? Die Deutsche Gesellschaft für Palliativmedizin sprach als Reaktion auf das Urteil von einem „gefährlichen Spielraum“, der diesen Organisationen nun geöffnet sei [14].

Warum sollten wir nach 5 Jahren mit einem verfassungswidrigen § 217 StGB ein (Straf‑)Gesetz brauchen, nachdem wir 150 Jahre ohne eine Strafnorm ausgekommen sind? Plausibel beantwortet wurde diese Frage noch immer nicht.

Während in anderen Ländern eine weitergehende Liberalisierungstendenz zu verzeichnen ist, bei der inzwischen sogar die aktive, direkte Sterbehilfe zugelassen wird, steht in Deutschland erneut die Frage einer Verschärfung des Strafrechts im Raum. Will man nicht den Fehler eines gesetzgeberischen Paternalismus wiederholen, einzelne Formen der Beihilfe unter Strafe zu stellen und letztlich wieder nicht erklären zu können, warum jene Form der Beihilfe strafbar sein müsse und jene nicht, dann kann sich eine Regelung schließlich nur auf die Gefährdung des autonomen Entschlusses beziehen.

## Rechtssicherheit?

Begonnen mit einem zu vernehmenden Wunsch nach Rechtssicherheit muss die Frage erlaubt sein, was mit Rechtssicherheit eigentlich gemeint ist. Solange das Strafgesetzbuch keine Strafbarkeit der Suizidbeihilfe kennt, ist diese nicht strafbar. Ein Strafbarkeitsrisiko wird bei einer möglichen Handlungspflicht und damit einer Strafbarkeit wegen Unterlassens nach Durchführung des Suizids gesehen. Dass der Suizidhelfer keine Rettungspflicht hat, ist weitgehend geklärt, auch wenn der Bundesgerichtshof es 2019 [15] versäumt hat, seine Wittig-Entscheidung^3^ aus dem Jahr 1984 [16] endgültig zu revidieren. Zu bedenken bleibt, ob eine Strafnorm nicht vielmehr sogar zu einem „unerwünschten Nebeneffekt“ führen könnte. Denn ein Anfangsverdacht, der ein Ermittlungsverfahren auslöst, könnte bei einer unnatürlichen Todesursache schnell angenommen werden, weil dieser sich aus objektiven Umständen ergibt [17].

## Wer leistet Beihilfe?

Damit stellt sich unweigerlich auch die Frage, wer als Suizidhelfer in Betracht kommt. Der strafrechtliche Begriff der Beihilfe ist zunächst einmal ein weiter und umfasst jede Handlung, die geeignet ist, die Haupttat (hier den Suizid) zu fördern. Das Interesse konzentriert sich in der Regel auf das tödlich wirkende Medikament, sodass sich der Blick auf die Ärzteschaft richtet. Auch wenn die Änderung der Musterberufsordnung der Ärzte zu verstehen gibt, dass die Ärzteschaft sich nicht als den primären Ansprechpartner sieht, sprechen dennoch viele Gründe für ihre Beteiligung, auch über das Verschreiben des Medikaments hinaus. Ärztliche Kompetenz wird immer dann gefordert sein, wenn es bei erkrankten Patienten mit Suizidwunsch um die Aufklärung mit Blick auf ihre Erkrankung und Behandlung, Linderung von Symptomlast, palliative Versorgung, Unterbreiten von Versorgungsangeboten sowie um Aufklärung im Hinblick auf die Wirkung des potenziell tödlichen Medikaments sowie ggf. auch mögliche Folgen bei Scheitern des Versuchs geht. Insbesondere die Personengruppe bestehend aus Patienten mit unheilbarer, tödlich verlaufender Erkrankung ist angesprochen und somit auch das Verhältnis zwischen behandelndem Arzt und Patient. Im Idealfall besteht hier ein Vertrauensverhältnis, in dem auch die Möglichkeit besteht, über Suizidgedanken zu sprechen, und der Arzt fühlt sich – ohne an strafrechtliche Konsequenzen denken zu müssen – imstande, dieses Gespräch offen zu führen. Dass innerhalb der Ärzteschaft durchaus eine grundsätzliche Bereitschaft besteht, Beihilfe zu leisten, ist aus Umfragen bekannt [18, 19]. Einiges spricht dafür, dem Arzt die Option der Suizidbeihilfe zu belassen. Er ist es, der den Patienten gut kennt, die Ambivalenz des Sterbewunsches einschätzen kann, den Patienten umfassend und individuell auf das Krankheitsbild zugeschnitten aufklären kann, ohne ihn unzulässig zu beeinflussen.

Schwieriger gestalten sich sodann solche Fallkonstellationen, in denen keine somatische, sondern eine psychische Erkrankung vorliegt. Gerade die Psychiatrie dürfte sich trotz ihrer Expertise rund um Fragen der Freiverantwortlichkeit schwer damit tun, einen Suizid zu unterstützen, wenn sich Suizidalität doch sonst als psychiatrischer Notfall definiert.

Erst recht dürfte es schwerfallen, einen Arzt für eine Suizidassistenz zu finden, wenn aus ärztlicher Sicht kein medizinischer Befund vorliegt, aber der Mensch des Lebens satt ist.

Sollen es also zukünftig Sozialarbeiter, Psychologen und Bürger in Form von ehrenamtlichen Suizidhelfern oder gar ein neu zu schaffender Beruf, der „Suizidhelfer“, sein, die die Beihilfehandlung erbringen?

## Zugang zur Suizidhilfe?

Diese Überlegungen werfen die Frage nach einem Zugang zur Suizidhilfe auf. Denn wer die Suizidhilfe für die eine Personengruppe erlaubt, kann diese einer anderen nicht versagen. Soll Hilfe Dritter in Anspruch genommen werden, scheinen vor allem zwei Wege denkbar: Das Aufsuchen einer anerkannten Beratungsstelle, die nach erfolgter Beratung einen „Beratungs- und Berechtigungsschein“ ausstellt, sodass ein tödlich wirkendes Medikament in Empfang genommen werden kann. Oder der Suizidwillige wendet sich mit diesem Anliegen an einen Sterbehilfeverein.

Zwei Gedankenstränge sollen einen Teil der jeweils damit verbundenen offenen Fragen an dieser Stelle lediglich anreißen: Wer sucht eine solche Beratungsstelle auf? Für die Personen mit psychischer Erkrankung stellt sich schnell die Frage, ob in der Beratungsstelle ausreichend Fachkompetenz vorhanden ist, um die Freiverantwortlichkeit zu beurteilen. Zudem stellt sich die Frage, wie eigentlich zu verfahren ist, wenn festgestellt wird, dass die Person nicht freiverantwortlich zum Suizid entschlossen ist. Löst dies eine Handlungspflicht aus? Könnte diese Überlegung Personen, insbesondere mit psychischer Erkrankung, abschrecken, diese Stellen aufzusuchen? Wie leicht zugänglich ist, ganz praktisch betrachtet, eine Beratungsstelle für ältere, auf Unterstützung anderer angewiesene Personen?

Verknüpft man nun aber das Beratungsangebot mit dem Ausstellen eines Scheins, der zum Erhalt eines tödlich wirkenden Medikaments berechtigt, kommen weitere Zweifel auf: Wie ist sichergestellt, dass das Medikament nicht bevorratet und sodann doch in einem Moment fehlender Freiverantwortlichkeit eingenommen wird? Dem Jahresbericht zum US-amerikanischen „Oregon Death with Dignity Act“, das einen ärztlich assistierten Suizid erlaubt, ist regelmäßig zu entnehmen, dass ein nicht unbeachtlicher Prozentsatz an Personen das Medikament gar nicht einnimmt [20]. Gedeutet wird dieses oftmals als eine Möglichkeit, einen Ausweg zu haben (im Englischen als „to be in control“), mit dem erklärt wird, dass es dem Menschen ausreiche, um die Möglichkeit der selbstbestimmten Lebensbeendigung zu wissen. Nun ist aber zu bedenken, dass Oregon die Beihilfe zum Suizid nur im Fall einer unheilbaren, bald tödlich endenden Erkrankung zulässt. Der Wunsch zu sterben wird somit oftmals höchst ambivalent ausfallen [21] und begleitet davon sein, den Rest des verbleibenden Lebens so lange zu leben, wie es – subjektiv empfunden – noch geht. Hier wird man wenig Zweifel an der Einschätzung der Freiverantwortlichkeit haben. Die Situation könnte sich aber bei Personen mit psychischer Erkrankung anders darstellen, die eben zum Zeitpunkt der Beratung psychisch stabil sind, sich aber das Medikament für eine erneute Verschlechterung ihrer Erkrankung bereits besorgt und damit bevorratet haben. Dennoch dürfte es keine gute Lösung für diese Fälle sein, dass die Person das Medikament in einem bestimmten Zeitfenster oder unter Aufsicht nehmen müsste, da damit ein unzulässiger, die Autonomie gefährdender Druck aufgebaut werden könnte.

## Kontrolle der Sterbehilfeorganisationen

Den Fokus auf Sterbehilfeorganisationen gerichtet, stellt sich oftmals die Frage nach Missbrauch oder fehlender Sicherung des freiverantwortlichen Willens (vgl. BVerfGE 153, 182, Rn. 222). Der bittere Beigeschmack rührt nicht selten aus dem Umstand, dass manche Sterbehilfevereine eine doch recht hohe „Aufwandsentschädigung“ oder einen „Mitgliedsbeitrag“ erheben, die das Eigeninteresse der Organisation/des Vereins zu belegen scheinen, oder es werden aus dem Ausland Suizidmethoden bekannt, die nicht zum Bild eines würdevollen Sterbens passen wollen [22]. Es besteht die nicht unberechtigte Sorge, dass durch einen in einem Pflegeheim agierenden Sterbehilfeverein die Freiverantwortlichkeit der Entscheidung durch unzulässigen oder auch unterschwellig empfundenen Druck, dieses „Angebot“ doch wahrzunehmen, gefährdet werden könnte. Fürchtet man hier nun aus nicht völlig von der Hand zu weisendem Grund eine Gefahr für die Freiverantwortlichkeit der Entscheidung, erscheint eine Regelung, die das Handeln der Sterbehilfeorganisation staatlicher Kontrolle unterzieht, als Option.

## Freiverantwortlicher Suizidentschluss

Damit ist die Prüfung der Freiverantwortlichkeit als nächster kritischer Punkt aufgerufen. Da eine Beihilfe zur Selbsttötung nur dann straflos ist, wenn der Suizidentschluss freiverantwortlich erfolgt, muss dieser Punkt im Mittelpunkt aller Überlegungen stehen. Wie kann sichergestellt werden, dass der Suizidentschluss auf einen autonomen, freien Willen zurückgeht? Dieses soll dann der Fall sein, wenn er auf Grundlage einer realitätsbezogenen, am eigenen Selbstbild ausgerichteten Abwägung des Für und Wider ohne unzulässigen Druck erfolge (BVerfGE 153, 182, Rn. 240). Der Suizidentschluss muss von einer gewissen Dauerhaftigkeit und inneren Festigkeit getragen sein.

Auch wenn das Recht auf Selbsttötung nicht auf das Vorliegen einer unheilbaren, tödlich verlaufenden Erkrankung beschränkt ist, soll noch einmal auf die zuvor genannte grobe Einteilung von Personengruppen mit Suizidwunsch eingegangen werden. Denn Zweifel an der Freiverantwortlichkeit können sich in unterschiedlicher Intensität äußern. Es wird angenommen, dass 90 % der Suizide nicht freiverantwortlich erfolgen und auf eine psychische Störung zurückgehen (BVerfGE 152, 182, Rn. 245), wenngleich diese hohe Zahl zunehmend bezweifelt wird [23]. Wer könnte aber die Gruppe sein, die eine Beihilfe zum Suizid anfragt? Sind es die somatisch unheilbar Erkrankten – dann dürfte Freiverantwortlichkeit vor allem durch Aufklärung, Beratung und Zuwendung ermöglicht werden. Hürden wie ein Gutachterverfahren, die Anhörung oder die Fallvorstellung vor einer Kommission sowie (lange) Wartefristen erscheinen für diese Gruppe eher unangebracht und unzumutbar. Hier geht es um Menschen, die – ohne Erkrankung – weiterleben wollen, es – wegen der Erkrankung – aber nicht können. Es darf in diesen Fällen nicht infrage gestellt werden, dass ein Mensch grundsätzlich fähig ist, eine freiverantwortliche Entscheidung, auch die zur Selbsttötung, zu treffen. Nur bei aufkommenden Zweifeln an der Freiverantwortlichkeit ist eine weitere Prüfung angezeigt. Diese vielleicht schon stark geschwächten Personen auf Beratungsstellen oder an Gutachterverfahren zu verweisen, erscheint mindestens dann unangemessen, wenn ein vertrauensvolles Arzt-Patienten-Verhältnis besteht. Da diese Personen in der Regel ärztlich begleitet werden, sie damit grundsätzlich ein Aufklärungs- und Beratungsangebot haben, ist die Gefahr einer unzulässigen Beeinflussung des Willensentschlusses eher gering. Der Gesetzgeber sollte sich also (weitgehend) zurückhalten.

Diffiziler erscheint die Betrachtung der Personengruppe mit (schweren) psychischen Erkrankungen. Eine freie Suizidentscheidung setzt die Fähigkeit voraus, seinen Willen frei und unbeeinflusst von einer akuten psychischen Störung zu bilden und nach dieser Einsicht handeln zu können (BVerfGE 153, 182, Rn. 241). Psychiatrische Expertise wird dabei oftmals gefragt sein [24]. Dabei scheinen Zweitmeinungen oder ein einzuholendes Gutachten überzeugender als die Anhörung durch eine Kommission [25], die kaum in der Lage sein dürfte, die Freiverantwortlichkeit in dem ihr zur Verfügung stehenden Zeitrahmen verlässlich zu prüfen. Zudem dürfte es diese Personengruppe schwer haben, einen zur Suizidhilfe bereiten Arzt zu finden. Probleme einer Beratungsstelle könnten aber hier wieder sein: Bevorraten eines Medikaments für die nächste Krise, Umgang mit suizidgefährdeten, nicht freiverantwortlichen Personen.

Es verleibt die dritte Gruppe, für die es eine Antwort zu suchen gilt: Personen, ohne (ernsthafte) Erkrankung, die ihres Lebens satt sind. Hierunter kann der „Bilanzsuizid“ (beschrieben als rationale Abwägung von Lebensumständen) fallen, die Lebensmüdigkeit einer älteren Person im Pflegeheim oder einer Person, die glaubt ihr Leben gelebt zu haben und es nun beenden möchte. Diesen Personen dürfte es derzeit wohl schwerfallen, außerhalb von Sterbehilfeorganisationen einen Suizidhelfer zu finden. Dennoch sind es gerade Personen in Pflegeheimen, ältere Personen sowie Personen, die einen geliebten Menschen verloren haben und nun ihm folgen wollen, bei denen die nicht von der Hand zu weisende Sorge besteht, dass auf diesen Menschen ein Druck lasten könnte, Suizidhilfe in Anspruch zu nehmen, weil sie ihre Lage als aussichtlos empfinden, finanzielle Sorgen sie wegen des teuren Pflegeheimes bedrücken oder sie sich als Last für andere sehen. Diese autonomiegefährdenden Faktoren sind ernst zu nehmen. Es ist eine große gesamtgesellschaftliche Frage, die den Umgang mit älteren, auf Hilfe anderer angewiesenen Menschen betrifft und auf die es dringend Antworten zu finden gilt. Es wäre jedoch ein fragwürdiges Verständnis von Suizidprävention, Suizide vor allem durch Verbote verhindern zu wollen [26]. Auch wenn die Vorstellung von Sterbehilfeorganisationen in einem Pflegeheim großes Unbehagen bereitet, darf das Recht auf ein selbstbestimmtes Sterben diesen Personen nicht abgesprochen werden. Gleichwohl dürften gerade auch die Beschränkungen bis hin zur Isolation und das Abschotten von den eigenen Angehörigen in Zeiten der COVID-19-Pandemie aufgezeigt haben, dass Personen in Pflegeheimen in vielerlei Hinsicht vulnerabel sind und in solchen Situationen ein Gefühl von absoluter Hilf- und Ausweglosigkeit entstehen kann. Umso wichtiger sind Verfahrenssicherungen. Neben dem Angebot von Beratungsstellen könnte eine Regelungsoption sein, Sterbehilfeorganisationen einer strengeren Aufsicht zu unterwerfen, sodass ihr Handeln staatlicher Kontrolle unterliegt. So müsste eine festgeschriebene Prüfung der Freiverantwortlichkeit einschließlich eines umfassenden Aufklärungs- und Beratungsangebots Bestandteil eines Verfahrens sein. Zudem dürfte die Prüfung der Freiverantwortlichkeit und die Suizidbeihilfe durch Verordnung eines Medikaments nicht von ein und derselben Person durchgeführt werden.

## Quo vadis, Gesetzgeber?

Der Blick auf verschiedene Personengruppen, die eine Suizidbeihilfe wünschen könnten, zeigt, vor welchen Schwierigkeiten der Gesetzgeber auf der Suche nach einer Regelung für alle Personen steht. Mit einer Regelung bewegt sich der Gesetzgeber im Spannungsverhältnis zwischen Autonomie- und Lebensschutz. Zwar darf er regeln, die Regelung darf aber nicht dazu führen, dass dem Einzelnen faktisch die Inanspruchnahme einer Suizidhilfe unmöglich gemacht wird. Hinter den unterschiedlichen Gesetzesvorschlägen und -entwürfen, die in der kommenden Legislaturperiode die weitere Debatte begleiten könnten, stehen unterschiedliche Regelungskonzepte [26–31]. Diese werden nicht nur unterschiedliche Vorstellungen von der Umsetzung des Rechts auf ein selbstbestimmtes Sterben widerspiegeln, sie haben auch vermutlich unterschiedliche Effekte und werden unterschiedliche Personen ansprechen. Gegenüber Verbotsnormen verdienen Aufklärungs- und Beratungskonzepte den Vorzug, weil sie die Person in ihrer grundsätzlichen Fähigkeit zur freiverantwortlichen Entscheidung anerkennen und Gefahren einer nicht freiverantwortlichen Entscheidung durch Aufklärung, Beseitigung von Fehlvorstellungen, der Unterbreitung von Alternativen etc. verringern können [3, 26].

Dennoch bleibt die Frage, ob eine Regelung geschaffen werden soll, die den Zugang aller zur Suizidbeihilfe eröffnet. Wer mit dem Prinzip Verbot-Ausnahme arbeitet, wird allerdings keine Gruppe von den Ausnahmen des Verbots ausschließen können. Eine Regelung darf nicht darauf zielen, durch Verbote die Anzahl an Suiziden gering zu halten. Dieses wäre aus verfassungsrechtlichen Gesichtspunkten ein falscher Ansatz einer Suizidprävention. Die grundsätzliche Vorzugswürdigkeit prozeduraler Absicherungen sollte kritisch auf die Eignung ihres jeweiligen Verfahrens geprüft werden. So dürften sich Kommissionen, z. B. ähnlich denen zur Präimplantationsdiagnostik [32], für die Entscheidung über eine Suizidbeihilfe als ungeeignet erweisen. Der Betroffene hat ein Recht auf selbstbestimmtes Sterben. Dieses Recht ist nicht infrage zu stellen oder einer ethischen Reflexion durch Dritte zu unterziehen. Sichergestellt werden muss nur, dass die Person alle für sie relevanten Informationen hat und auf dieser Basis freiverantwortlich entscheidet.

Konzepte, die auf Aufklärung und Beratung setzen, können für den Menschen mit Suizidwunsch niedrigschwellig wirken und damit den Zugang zur Beratung ermöglichen, die ergebnisoffen, aber auf den Erhalt des Lebens orientiert sein kann. Diese Konzepte ermöglichen es, das Motiv für einen Suizid zu erfragen, ohne das Motiv zu bewerten, und im Hinblick auf das Motiv passende Alternativen anzubieten. Sie ermöglichen auch eine erste Prüfung, ob die Person über alle relevanten Informationen verfügt, ob sie diese erfassen, bewerten und für sich abwägen kann. Damit ist keine Überhöhung der Autonomie des Einzelnen verbunden, sondern ein Anerkennen, dass jede Person grundsätzlich über die Fähigkeit verfügt, eine autonome Entscheidung zu treffen. Gleichwohl muss hinterfragt werden, ob nicht insbesondere Personen mit unheilbarer, tödlicher Erkrankung die Unterstützung besser durch einen Arzt erhalten könnten. Der Vorteil eines niedrigschwelligen Angebots könnte sich für bestimmte Personen jedoch als Nachteil erweisen, weil der Zugang zu Suizidmitteln zu leicht wird. Die Überlegung bleibt daher bestehen, ob eine Regelung getroffen werden sollte, die jedem den Zugang zum Suizidmittel ermöglicht oder ob man besser insbesondere von Verboten Abstand nehmen sollte.

Die Überlegung, Beratungsstellen oder Kommissionen einzurichten, rührt auch aus der Haltung der Ärzteschaft, die im Rahmen ihrer Berufsordnung kein Verfahren anbietet, das die Gewissensentscheidung des Einzelnen anerkennen würde. Erneut bringt die Bundesärztekammer mit ihrer Musterberufsordnung zum Ausdruck, dass die Ärzteschaft für diese Art von Wünschen nicht zuständig sei. Dies spiegelt weder den Wertepluralismus innerhalb der Ärzteschaft wider noch bietet es eine Lösung für das bestehende Problem an. Zugleich werden Gesetzgebungsvorschläge unterbreitet, die durchaus die Ärzteschaft in den Fokus nehmen [27, 28, 30, 31], sei es in ihrer Rolle als behandelnder Arzt, als Gutachter für die Frage der Freiverantwortlichkeit, als Aussteller des begehrten Rezepts. Doch wird man der Ärzteschaft kaum diese Verantwortung zuschreiben können, wenn sie diese nicht annehmen will. Da niemand zur Suizidhilfe verpflichtet ist, könnte es ins Leere laufen, ausgerechnet die Ärzteschaft als Suizidhelfende auszuwählen.

## Schlussbemerkung

Eine Regelung, die alle Fallkonstellationen in den Blick nimmt und angemessen differenziert, ohne das Suizidmotiv des Einzelnen zu bewerten, wird berücksichtigen müssen, dass der weit überwiegende Teil aller Suizid(-versuche) nicht freiverantwortlich erfolgt. Deshalb müssen bei einem Zugang zur Suizidbeihilfe entsprechende Verfahrenssicherungen eingeführt werden, jedenfalls wenn nach Durchlaufen einer Beratung unmittelbar der Zugang zum Suizidmittel eröffnet wird. Verfahrensrechtliche Hürden wie Gutachterlösungen, betreuungsrechtliche Verfahren, verschiedene Überlegungen zu Kommissionen (mit Votum) oder das Aufsuchen bestimmter Beratungsstellen könnten sich für bestimmte Personengruppen, v. a. für unheilbar kranke Menschen, die bald sterben werden, als unzumutbare und gar unmenschliche Maßnahmen erweisen. In vielen dieser Fälle würde die Inanspruchnahme der Hilfe Dritter zur Selbsttötung sogar verunmöglicht bzw. faktisch unmöglich werden. Dabei stand doch bisher gerade diese Gruppe im Fokus der Überlegungen. Es bleibt daher ernsthaft zu überlegen, ob es gelingen kann, diese höchst intime, private und existenzielle Frage durch ein Gesetz, das allen Fallgruppen gerecht werden soll, zu regeln. Der Schutz einer hoch vulnerablen Personengruppe sollte jedenfalls nicht zulasten einer anderen erfolgen. Vor diesem Hintergrund sollte der Gesetzgeber von einer Neuregelung eines § 217 StGB Abstand nehmen.

## Footnotes

1 Überdies wird für eine straflose Beihilfe zur Selbsttötung verlangt, dass der Suizident die letzte, zum Tode führende Handlung selbst ausführt. Die sog. Tatherrschaft über das Geschehen („point of no return“) spielt für die Abgrenzung zur Tötung auf Verlangen (§ 216 Strafgesetzbuch [StGB]) eine Rolle. Die Tötung auf Verlangen soll jedoch nicht Gegenstand dieses Beitrags sein.

2 § 217 Geschäftsmäßige Förderung der Selbsttötung. (1) Wer in der Absicht, die Selbsttötung eines anderen zu fördern, diesem hierzu geschäftsmäßig die Gelegenheit gewährt, verschafft oder vermittelt, wird mit Freiheitsstrafe bis zu drei Jahren oder mit Geldstrafe bestraft. (2) Als Teilnehmer bleibt straffrei, wer selbst nicht geschäftsmäßig handelt und entweder Angehöriger des in Absatz 1 genannten anderen ist oder diesem nahesteht (gem. BVerfGE vom 26.02.2020 verfassungswidrig und nichtig).

3 In dem als Wittig-Entscheidung bekannt gewordenen Fall ging es um die Frage der Rettungspflicht des Arztes gegenüber einer Patientin, die er bewusstlos nach einem Selbsttötungsversuch auffindet.
